# Breeding for climate adaptation: genetic variation and genomic selection for drought response in Scots pine

**DOI:** 10.1186/s12864-026-12849-x

**Published:** 2026-04-27

**Authors:** Rajiv Chaudhary, Maximiliano Estravis Barcala, Irena Fundova, Tomas Funda, Zhi-qiang Chen, Harry X. Wu

**Affiliations:** 1 Department of Forest Genetics and Plant Physiology, Umeå Plant Science Centre (UPSC), Swedish University of Agricultural Sciences, Umeå, Sweden; 2 State Key Laboratory of Tree Genetics and Breeding, Co-Innovation Center for Sustainable Forestry in Southern China, College of Forestry, Nanjing Forestry University, Nanjing, 210037 China; 3 Norwegian Institute of Bioeconomy Research (NIBIO), Høgskoleveien 8, Ås, 1433 Norway

**Keywords:** Drought, Climate adaptation, Dendroecology, GBS, Genomic selection, Scots pine

## Abstract

**Background:**

Drought intensity and frequency are increasing under global warming in the boreal forests, and breeding for drought resistance will facilitate adaptation of new planting material to changing climate conditions. We used a tree-ring dataset of 559 individuals to study Scots pine genetic variation and the efficiency of genomic selection of drought-response traits (drought resistance, recovery and resilience), for the first time. From genotyping-by-sequencing (GBS), 31,101 SNPs were generated and used for the study.

**Results:**

Significant genetic variation was detected for drought-response and other growth, wood-anatomy and wood density traits. Heritability estimates for wood-anatomical traits were higher than those for drought-response and growth traits. Genetic correlations between drought-response and wood-anatomical traits were generally high but mostly nonsignificant. In contrast, drought resistance and recovery showed positive and significant correlations with basal area increment and height. We found that the predictive ability and accuracy for drought-response traits were lower than those for wood-anatomical traits, and were comparable between GBLUP and ABLUP. Greater genetic gain per year can be achieved through genomic selection relative to pedigree-based selection if the generation interval is reduced.

**Conclusions:**

The positive genetic correlation between drought-response and growth traits will enable simultaneous selection for improved growth and increased drought resistant trees in Scots pine breeding through either pedigreed-based and genomic selection.

**Supplementary Information:**

The online version contains supplementary material available at 10.1186/s12864-026-12849-x.

## Background

The intensity and frequency of regional droughts in the boreal forests of Northern Hemisphere have increased drastically during recent decades under global warming [1, 2]. Scandinavian countries such as Sweden have experienced summer temperatures up to 5° warmer than the long-term average [3]. A variety of climatic factors, such as rising temperature, shifting precipitation patterns, and increased evaporative demand under drought stress, limit the radial growth of trees [4–6], influence carbon flux capacity [7, 8], reduce the survival rate and tree productivity [9, 10]. The rapid changes in climate threaten the ability of trees to cope with new environmental conditions [11–13], while also increasing their vulnerability to pathogens, insects, and other disturbances [14–16]. It is important to understand the adaptive responses of forest tree species to future changing climatic regimes for guiding assisted migration or breeding for adaptation [11, 13, 17]. Many studies have shown the intraspecific genetic variation in growth and drought-response traits among provenances and progeny field trials under rapidly changing climate [18–21]. Such genetic variation in drought response can be exploited in tree breeding and reforestation programmes to improve resilience for future planted stock [22].

Tree height (HGT) and diameter at breast height (DBH) have been the main focus of genetic improvement in conifer tree species in the past [23]. In Sweden, intensive genetic improvement programmes for DBH and HGT have been established since the 1950s [24, 25]. Wood quality traits such as wood density (WD), microfibril angle (MFA), and modulus of elasticity (MOE) have been estimated in Norway spruce and Scots pine [26, 27]. However, the evaluation of traits associated with climate adaptation, such as drought resistance, remains very limited and labour-intensive [28], making it difficult to predict the adaptive capacity of tree species [13, 29]. Common garden experiments have demonstrated signals of local adaptation in tree species [19, 30–32], and have also revealed local genetic adaptation of drought-stress response in a white spruce (*Picea glauca* (Moench) Voss) provenance trial [19]. Several studies have evaluated tree growth resilience in response to extreme drought episodes [33–35]. With rapid climate change, it is difficult to predict how forests will resist drought and recover after such events. Therefore, tree breeders need to incorporate new drought-response traits into selection and breeding scenarios.

Dendroecology is the study of annual growth rings in trees, and variation in these rings provides retrospective insights for detecting tree growth-climate relationships throughout the lifespan of trees [19, 31, 36, 37]. Tree-ring data provide three main categories of phenotypic traits that can serve as proxies for tree vulnerability to drought: (a) wood-anatomical traits, (b) drought-response traits (DR traits), and (c) climate-sensitivity traits (CS traits) (Table 1). Wood-anatomical traits (e.g., radial conduit wall reinforcement (CWRr) and radial lumen diameter (LDR)) allow the assessment of the hydraulic function of the xylem and act as screening traits for drought sensitivity [33, 38–40]. Basal area increment (BAI) is a good predictor of survival, as trees growing at a slower rate are more vulnerable to drought [19, 41, 42]. DR traits describe the capacity of trees to withstand drought conditions (resistance), to recover after drought events (recovery), and to regain pre-drought growth levels (resilience) [1, 18, 19, 41]. CS traits capture the overall effect of drought across the life span of a tree, detecting climatic constraints affecting growth [19, 31]. Some breeding programmes have already integrated resilience as an abiotic factor in conifer tree species [43–45] and evaluated the efficiency of genomic prediction for drought response in the breeding population of white spruce [22].

**Table 1:** List of abbreviations and definitions of tree-ring traits and climatic variables

Variable name	Unit	Description
Growth traits		
HGT	m	Height
DBH	cm	Diameter at breast height
Wood-anatomical traits		
RW	mm	Ring width
BAI	mm^2^	Basal area increment
WD	kg/m^3^	Wood density
LDR	µm	Lumen diameter in the radial direction
LDT	µm	Lumen diameter in the tangential direction
LD	µm	Lumen diameter
CWT	µm	Cell wall thickness
CWR	unitless	Average conduit wall reinforcement
CWRr	unitless	Radial conduit wall reinforcement
Drought response traits		
RS	unitless	Resistance of a tree in response to a periodic drought
RC	unitless	Recovery of a tree in response to a periodic drought
RL	unitless	Resilience of a tree in response to a periodic drought
Climate sensitivity traits		
CS	unitless	Climate sensitivity traits. Coefficient of correlation between a wood trait X and a climatic variable in month Y (month of the previous year and the current year are labelled (t − 1) and (t), respectively)
Climatic variables		
MAT	°C	Mean annual temperature
MAP	mm	Mean annual precipitation
SPI	index	Monthly standardized precipitation index
SPEI	index	Monthly standardized potential evapotranspiration index

Scots pine (*Pinus sylvestris* L.) is the second most widely distributed conifer species in the Northern Hemisphere and an important ecological and economic tree species for the global timber and pulp industry. Episodes of drought have caused severe mortality in Scots pine over the past two decades [9, 46–48]. Assessing its adaptive capacity to future climates is therefore of great importance to prevent large-scale maladaptation to local conditions and associated forest decline. Scots pine populations have been reported to be locally adapted to drought in relation to tree height [49, 50]; shoot morphology [51]; phenology [52].

Recent advancements in genomics have accelerated tree breeding approaches, with increasing efforts focusing on genomic prediction using dense marker panels [27, 45, 53]. Conifer breeding typically requires 6–15 years of progeny testing to select optimally adapted seedlings. Genomic selection (GS) is a promising strategy that substantially shortens breeding cycles by allowing selections from 1-year‐old seedlings, without the need to phenotype the traits [54]. This would increase the selection intensity, leading to higher genetic gains per unit time [55, 56]. In the training phase, GS requires both phenotypic and genotypic data using a training population to develop prediction models, which are then applied to predict genomic estimated breeding values (GEBVs) in a validation population. In the validation phase, only genotypic data are required to obtain GEBVs using the prediction model developed in the training phase [44, 54]. GS has been successfully applied in forest trees to predict growth and wood quality [57–60], and GS models for Norway spruce and Scots pine have reached almost the same efficiency as the conventional breeding [26, 27]. However, GS for DR and novel wood-anatomical traits have been explored in a few studies only [22, 57].

In the current study, we used a tree-ring dataset from a Scots pine progeny trial to provide new tree-ring phenotypes and to evaluate genetic variation in drought-response parameters (DR traits) as well as the efficiency of genomic predictions. Tree-ring data from the Grundtjärn site had previously been used to evaluate the efficiency of genomic predictions for growth and wood-quality traits such as HGT, DBH and WD [26] based on genotyping-by-sequencing (GBS) [61], with reads aligned against *Pinus taeda* v1.0 reference genome to detect SNPs [62]. In the present study, we used the first Scots pine reference genome (under review) to detect SNP markers. The main objectives were: (i) to detect tree growth-climate relationships among 158 Scots pine families; (ii) to compare the efficiency of genomic versus pedigree estimation for DR traits together with the wood-anatomical and growth traits; and (iii) to evaluate the theoretical accuracy of genomic predictions in one of the Northern Swedish breeding population of Scots pine.

## Methods

### Study site, biological material and experimental design

The Grundtjärn progeny trial used in this study consisted of full-sib families of Scots pine, established in 1971 by the Forestry Research Institute of Sweden (Skogforsk) in Northern Sweden (#S23F 711261, latitude 63.55˚N, longitude 17.41˚E, elevation 320 m, area 3.5 ha) [26, 63–65]. Samples were collected from 559 Scots pine trees representing 158 full-sib families generated with a partial diallel mating design using 33 parents, where each parent participated 8–9 times either as a mother or a father. The mating design of this progeny test was described in detail by Fries [65]. Briefly, the experimental design consisted of one-year-old seedling planted at 2.2 × 2.2 m spacing in a completely randomized single-tree plots design, as described in Fundova et al. [63, 64]. The parental (plus) trees of these progenies originated from natural forest populations between latitudes 63°NE and 64°NE and were selected for their outstanding phenotypic values. The number of trees per family varied from one to seven (Table S1).

### Climate variables

Mean annual temperature (MAT), mean annual precipitation (MAP), monthly standardized precipitation index (SPI) [66] and monthly standardised potential evapotranspiration index (SPEI) [67] were extracted using the Climate Downscaling Tool (ClimateDT) (approx. 1 km^2^) (https://www.ibbr.cnr.it/climate-dt/) [68] for the Grundtjärn site. SPEI and SPI indices were calculated in R using the “SPEI” package v. 1.8.0 [69] (Method S1). Interpolated climate data for the site covered the period 1990–2011. Details on climatic variables and their abbreviations are reported in Table 1.

### Phenotyping of growth, wood-anatomical and drought-response traits

The phenotypic data consisted of growth, wood-anatomical, drought-response (DR) and climate-sensitivity (CS) traits (Table 1). Growth traits were measured on each tree in 2011, 40 years after the progeny trial establishment. Ten-millimeter increment cores were extracted at breast height (1.3 m above ground) from the southeastern side of each stem and processed as described in Fundova et al. [63, 64]. Wood-anatomical data were obtained using the SilviScan technology (CSIRO, Australia). Cross-dating of the 559 individual tree-ring series was validated in R using the dplR package v. 1.7.4 [70]. Wood-anatomical data included measurements of annual ring-widths (RW), cell wall thickness (CWT), radial lumen diameter (LDR), tangential lumen diameter (LDT), and wood density (WD). RW was further transformed into BAI using the “bai.out” function in the dplR package [70]. Conduit-wall reinforcement (CWR) was obtained from wood traits using the formula CWR = (CWT/LD)² as described in [19], where LD is the average of LDR and LDT. DR traits [41], namely resistance (RS), recovery (RC), and resilience (RL), were derived from BAI using the “pointres” R package v. 2.0.2 [71]. The calculation of DR traits is shown in Method S1. The RS is the capacity of trees to withstand drought and resist growth reduction. RS was estimated as DR/preDR, where DR and preDR are the ratio between BAI during and before the drought period, respectively. The RC is the ability of a tree to recover its growth after drought period. RC was estimated as postDR/DR, where postDR and DR are the ratio between BAI after and during the drought period, respectively. Resilience (RL) is the ability of tree to reach pre-drought growth levels after drought. RS was calculated as postDR/preDR [1, 18, 19, 41]. CS traits (COR_*X*–*Y*(*t*)_) are Pearson correlation coefficients between mean residual chronologies and a given climate variable.

### Dendroclimatic analysis

**Fig. 1:**
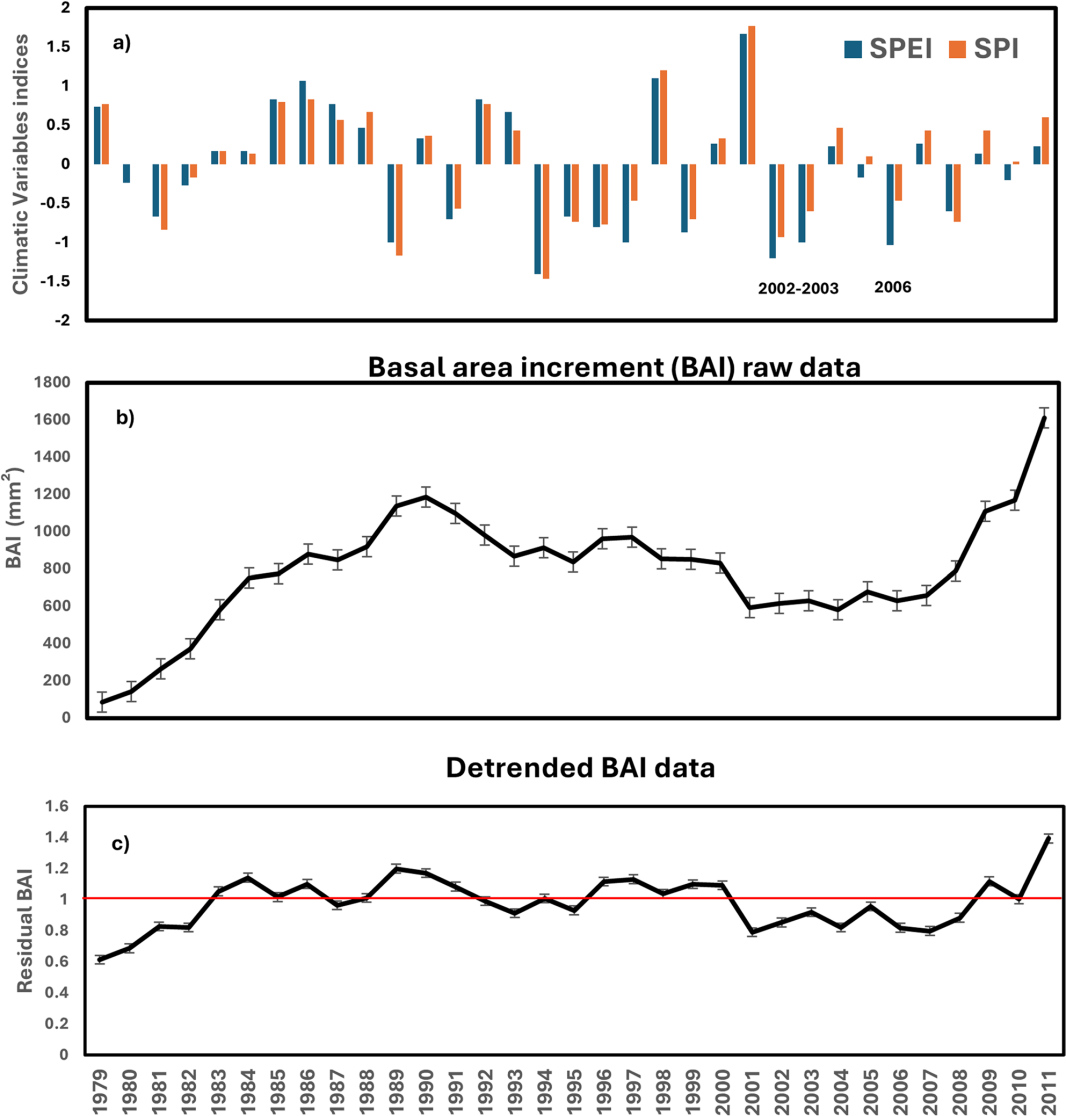
Climate variables and mean basal area increments (BAI) indices of Scots pine families from 1979 to 2011 at the Grundtjärn. **a** Climatic variables of mean SPEI and SPI (July to September). Positive and negative values of SPEI and SPI represent wetter and drier conditions, respectively. Years 2002 − 2003 and 2006 indicate drought years. **b** Mean annual variation in BAI. **c** Mean detrended BAI (index) for the period 1979 − 2011. Standard deviations are presented by error bars

Dendroclimatic analyses were conducted to assess the relationship between wood anatomical traits and climatic variables at the Grundtjärn site. First, raw data of wood-anatomical traits were detrended with a “spline curve model” to eliminate non-climatic factors related to age- and size-related growth trends, using the dplR package [70]. Second, first-order autoregressive (AR) model was used to remove autocorrelations present in the time series of wood traits from individual cores, also using dplR [70]. The resulting residuals were averaged to construct mean standardized residual chronologies for each family, using a robust bi-weight mean to reduce the effect of outliers. Data from 1976 to 1989 were excluded to avoid the early juvenile growth stage, when age and size strongly affect Scots pine growth (Fig. 1). Finally, the R package treeclim v. 2.0.6.0 [72] was used to examine dendroclimatic relationships between the residual chronologies of wood-anatomical traits and monthly climate variables at the Grundtjärn site for the period of 1990–2011, applying the “dcc” function with a bootstrap approach.

### DNA isolation and genotyping

Total genomic DNA was extracted from vegetative buds or needles from 559 Scots pine samples using the commercial NucleoSpin^Ⓡ^ Plant II kit (Machery-Nagel, Dren, Germany), following the manufacturer’s instructions. Extracted DNA concentration was measured with Qubit^Ⓡ^ 2.0 fluorometer (Invitrogen, Carlsbad, CA, USA). Three genomic libraries for GBS were prepared as described in [26, 73] using PstI high fidelity restriction enzyme (New England Biolabs, MA, USA). The libraries were sequenced on an Illumina HiSeq 2000 platform at SciLifeLab, Sweden.

### Bioinformatic analysis and variant calling

Demultiplexing of GBS reads was done using Stacks v1.40 [74]. Quality control was performed with FastQC v0.11.9 [75] and summarized with MultiQC v1.12 [76] previously described in Calleja-Rodriguez et al. [26]. Clean, paired reads were mapped to the *P. sylvestris* reference genome (under review) using STAR v2.7.11a [77] with default parameters, choosing sorted BAM as output. All samples were jointly called by BCFtools v1.19’s [78] mpileup command with default parameters. The variant filtering process is illustrated in Fig. S1. First, variants in masked regions of the genome were discarded. Variants with genotyping quality lower than 30, missing in more than 40% of individuals, or at 5 or less base pairs from an indel, were filtered out. Finally, indels were discarded, and only biallelic SNPs with MAF (minor allele frequency) higher than 5% were kept. This brought the final number of SNPs to 33,000. VCFtools v0.1.16 [79] was used for variant manipulation and filtering. The missing genotypic data were imputed using LDkini [80] implemented in TASSEL v. 5.2.96 [81]. In total, 11.6% of missing genotypes were imputed. Second filtering was done using the qc.filtering function in the R package ASRgenomics v. 1.1.4 [82], and after quality filtering step, 31,101 informative SNPs were retained, which were used for GS analysis.

### Statistical analysis

Initially for HGT and DBH in the progeny trial, univariate single site spatial analysis were performed to reduce the within-trial micro-environmental effects from the raw data as described in the study of Calleja-Rodriquez et al. [26]. The environmentally adjusted phenotypic data (predicted values) were used for the genetic analysis. Other phenotypic traits were detrended to remove systematic non-genetic trends prior to fitting genomic selection models using spline curve and AR model to improve prediction performance in genomic selection analyses.

For each trait, an individual-tree linear mixed model was fitted in ASReml-R v.4.2 [83] using the additive genomic relationship matrix (**G matrix**) and the pedigree-based relationship matrix (**A matrix**) to estimate additive variance, heritability, and breeding values. The following model was used for GBLUP (genomic best linear unbiased predictions) and ABLUP (pedigree-based best linear unbiased predictions):

1 $$\:\begin{array}{c}y=X\beta\:+W\alpha\:+e\end{array} $$

where ***y*** is the vector of phenotypic data for each trait, **β** is a vector of fixed effects for the overall mean, **α** is the vector of random additive genetic effects, that is assumed to follow a normal distribution with expectations of ***α*** ~ *N* (**0**, σ^2^_α_ **G**) or ***α*** ~ *N* (**0**, σ^2^_α_ **A**) for GBLUP and ABLUP, respectively, and ***e*** is the vector of residual effects, where ***e*** ~ *N* (**0,** σ^2^_e_**I**). σ^2^_α_ is the additive genetic variance and σ^2^_e_ is the residual variance. ***X*** and ***W*** are the incident matrices of **β** and** α**, and the **I** matrix is an identity matrix. The function “G.matrix” from the ASRgenomics package was used to generate the **G** matrix [82] for additive genetic relationships, which is equivalent to the method described by VanRaden [84]. The **G** matrix was tuned up to ensure stability for matrix inversion and downstream analysis. Finally, the “G.inverse” function from the ASRgenomics package was used to obtain the inverse of **G** matrix. The inverse of **A** matrix was generated using the function “ainverse” from the ASReml-R package. The **G**^− 1^ matrix for GBLUP was replaced by **A**^− 1^ matrix for the ABLUP method.

### Narrow-sense heritability

For each trait, narrow-sense heritabilities $\:\widehat{h}\:{^{2}}$ were estimated for ABLUP and GBLUP as:   

2 $$\:\begin{array}{c}{\widehat{h}}^{2}=\frac{{\widehat{{\upsigma\:}}}^{2}_a}{{\widehat{{\upsigma\:}}}^{2}_a + {\widehat{{\upsigma\:}}}^{2}_e}\end{array}$$

We used the “vpredict” function of the ASReml-R package to estimate standard errors of the heritabilities.

### Cross validation and estimation of prediction efficiency

To evaluate the prediction efficiencies of the ABLUP and GBLUP, a 10-fold cross-validation (CV) analysis was performed for all traits with 10 replications. The progeny data were randomly divided into ten-folds, of which nine were used as the training population (TP) (90%) and one as the validation population (VP). In each CV round, phenotypes and breeding values were predicted for the remaining fold (10%), with each analysis replicated ten times using the individual tree model in Eq. 1.

Prediction efficiencies of ABLUP and GBLUP were evaluated and compared based on the predictive ability (PA) and predictive accuracy (PAC), which are the most common metrics used in tree breeding [22, 26, 85–86].

The PA was calculated as the Pearson correlation coefficient between the predicted GEBVs or pedigree-based breeding values (EBVs) of the individuals in the VP and their adjusted phenotypes (*y*),

$$\mathrm i.\mathrm e.,\;\mathrm{PA}=\mathrm{corr}\left({\mathrm{EBV}}_{\mathrm{VP},\mathrm y}\right)\mathrm{or}\;\mathrm{PA}=\mathrm{corr}\left({\mathrm{GEBV}}_{\mathrm{VP},\mathrm y}\right).$$

PAC was estimated as:

3 $$\:\begin{array}{c}PAC=\raisebox{1ex}{$PA$}\!\left/\:\!\raisebox{-1ex}{$\sqrt{{h}^{2}}$}\right.\end{array}$$

where √h² is the square root of individual-tree narrow-sense heritability obtained from Eq. 2, and PA is the predictive ability. We used the √h² estimated from GBLUP method as our best estimate of heritability to calculate PAC for both ABLUP and GBLUP methods.

Theoretical accuracy (TAC) was estimated using GBLUP only.

4 $$\:\begin{array}{c}{TAC}_{VP}=\sqrt{1-{SE}_{\:\:i\:}^{2}/\left(\left(1+{F}_{i}\right){\widehat{{\upsigma\:}}}_{\:\:{\upalpha\:}\:}^{2}\right)}\end{array}$$

where *SE*_*i*_ is the standard error of the breeding value of the VP, *F*_*i*_ is the inbreeding coefficient of the i^th^ individual, and 1 + *F*_*i*_ is the diagonal element of the i^th^ individual in the **G** matrix.

For each trait, estimations were performed for the evaluation methods within each fold and averaged across folds and replicates. The estimation of TAC has been described to evaluate the efficiency of prediction models in other studies [87, 88].

### Estimation of ABLUP accuracy without cross-validation as the benchmark of GBLUP accuracy

GBLUP was evaluated using ten-fold CV to assess theoretical accuracy (TAC). In contrast, ABLUP was fitted using the full pedigree and phenotypic dataset to evaluate TAC without a CV, derived from prediction error variances ensuring its comparability with GBLUP results. Both accuracies were derived analytically and reflects the expected reliability. This represents standard practice for evaluating pedigree-based models in animal and forest breeding and provide a practical alternative to CV for pedigree-based models [87, 89].

The TAC for ABLUP was estimated without using any CV method. The TAC estimated for ABLUP, for each individual and each trait, was calculated as:

5 $$\:\begin{array}{c}{TAC}_{i}=\sqrt{1-{SE}_{\:\:i\:}^{2}/\left(\left(1+{F}_{i}\right){\widehat{{\upsigma\:}}}_{\:\:{\upalpha\:}\:}^{2}\right)}\end{array}$$

where *SE*_*i*_ is the standard error of the breeding value of the i^th^ individual, *F*_*i*_ is the inbreeding coefficient of the i^th^ individual, and 1 + *F*_*i*_ is the diagonal element of the i^th^ individual in the **A** matrix.

### Phenotypic and genetic correlations between traits

To estimate genetic and phenotypic correlations between DR, wood-anatomical, and growth traits, a bivariate model was run, which was identical to the univariate model (Eq. 1) by stacking up the vectors for the two traits in ASReml-R. The following model was fitted:

6 $$\:\begin{array}{c}\left(\genfrac{}{}{0pt}{}{yi}{yj}\right)=X\beta\:\left(\mathrm{t}\right)+W\alpha\:\left(\mathrm{t}\right)+e\end{array}$$

where ***yi*** and ***yj*** are the stacked vectors of phenotypic values for traits *i* and *j*, respectively. **β(t)** is a vector of fixed effects including the mean for each trait, **α(t)** is the random additive effect within trait, with** α(t) **~ N(**0**, **Va** ⨂ **G**) for GBLUP, and **e** is the residual error, with **e **~ N(**0**, **Ve** ⨂ **I**). The matrix **G**^− 1^ (GBLUP) was replaced by **A**^− 1^ for (ABLUP) for the random additive genetic effect. The **I** matrix is an identity matrix. The matrices **Va** and **Ve** are 2 × 2 variance–covariance matrices, defined by the correlation between all pairs of traits ($\:\widehat{r}_{a}$ and $\:\widehat{r}_{e}$, respectively) and unique variances for each trait (i.e., CORGH in ASReml-R). The genetic correlation between traits was directly estimated from $\:\widehat{r}_{a}$ and the phenotypic correlation $\:\widehat{r}_{p}$ was calculated as:

7 $$\:\begin{array}{c}{\widehat{r}}_{p\left(i,j\right)}=\frac{{\widehat{COV}\left(i,j\right)}_{p}}{\sqrt{{\widehat{{\upsigma\:}}}^{2}pi{\widehat{{\upsigma\:}}}^{2}pj}}=\frac{\widehat{r}{\upalpha\:}\sqrt{{\widehat{{\upsigma\:}}}^{2}ai{\widehat{{\upsigma\:}}}^{2}aj}+\:\widehat{r}\mathrm{e}\sqrt{{\widehat{{\upsigma\:}}}^{2}ei{\widehat{{\upsigma\:}}}^{2}ej}}{\sqrt{\left({\widehat{{\upsigma\:}}}^{2}ai+{\widehat{{\upsigma\:}}}^{2}ei\right)\:\left({\widehat{{\upsigma\:}}}^{2}aj+{\widehat{{\upsigma\:}}}^{2}ej\right)}}\end{array}$$

where $\:\widehat{Cov}(i,j)_p$ is the phenotypic covariance between traits, and $\:\widehat{\upsigma}^{2}_{pi}$_,_ $\:\widehat{\upsigma}^{2}_{ai}$_,_ $\:\widehat{\upsigma}^{2}_{ei}$_,_ $\:\widehat{\upsigma}^{2}_{pj}$_,_ $\:\widehat{\upsigma}^{2}_{aj}$_,_ and $\:\widehat{\upsigma}^{2}_{ej}$ are the estimated phenotypic, additive, and residual variances of trait *i* and trait *j*, respectively. A likelihood-ratio test with one degree of freedom between the full model (Eq. 5) and a reduced model, assuming $\:\widehat{r}_{a}$ = 0 (a diagonal **V**_**a**_ matrix), was used to test the significance of the genetic correlation, and a likelihood-ratio test with two degrees of freedom, assuming no correlation between traits (i.e. $\:\widehat{r}_{a}$ = 0, $\:\widehat{r}_{e}$ = 0), was used to test the significance of the phenotypic correlation.

### Relative selection response of GS

The estimation of relative selection response (RSR) was obtained as a ratio of GS through ten-fold CV and the traditional pedigree-based selection (PS) without using any CV method estimated with the full population’s phenotypes and pedigree for the TAC and the breeding cycle time in years.

8 $$\:\begin{array}{c}{RSR}_{GS:PS\:=}\frac{{r}_{GS}}{{r}_{PS}}\times\:\frac{{CL}_{PS}}{{CL}_{GS}}\end{array}$$

where *r*_GS_ and *r*_PS_ are the efficiency of GS and PS, respectively, and *CL*_PS_ and *CL*_GS_ are the breeding cycle lengths of PS and GS, respectively. The selection efficiency of GS was estimated as (*RSR*_*GS*:*PS*_ − 1) × 100%. Two GS approaches were employed to estimate the relative *RSR*_*GS*:*PS*_, assuming a shortened breeding cycle, as described in Calleja-Rodriquez et al. [26]. Briefly, in the first approach, the breeding cycle was reduced to 18 years, as the flowering starts at 15–18 years in Scots pine, thereby shortening the progeny test time [90], whereas in the second approach, the breeding cycle was reduced to 11 years considering that the greenhouse stimulation of flowering [91] can induce female flowers at around 11 years. The breeding cycle for Scots pine using backward selection generally takes up to 36 years, while forward selection takes up to 21 years. We applied the two GS approaches in both backward and forward selection. Backward selection reduced the breeding cycle from 36 to 18 years and further to 11 years with flowering stimulation. Similarly, forward selection reduced the cycle from 21 to 18 years and subsequently to 11 years. RSR of GS (*RSR*_*GS*:*PS*_) was calculated using the TAC and both breeding cycle reduction approaches.

## Results

### Performance evaluation of families

Climate data revealed the occurrence of three drought episodes (2002, 2003, and 2006) (Fig. 1a) at the Grundtjärn site, and there was a notable decrease in BAI during the major drought events (Fig. 1b, c). The DR traits were derived from average values for 2002–2003, when drought conditions were most intense. For all the wood-anatomical and DR traits, substantial variability existed among the family means, and significant differences were observed among the families (*P* < 0.001; Table S1; Fig. S2). During the drought period, the most resistant families (34 and 147), which showed superior growth performance for BAI, also exhibited better resilience, but below-average recovery rate (Fig. S2). Most of the families (80, 118 and 138) with weaker radial growth recovered relatively well from the drought (Fig. S2).

### Climate–wood-anatomical traits relationship

**Fig. 2:**
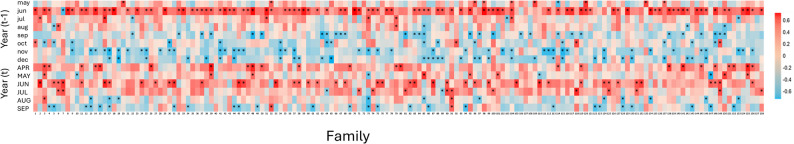
Climate–growth associations of Scots pine families. Correlation analyses of BAI residual chronologies and monthly SPEI at the Grundtjärn site from May to December of the previous growing season (t − 1) and April to September of the current growing season (t) over the 1990 − 2011 period. Months in capital letters represent the current year of ring formation, and months in lowercase represent the preceding year. The scale bar indicates positive (red) and negative (blue) correlation coefficients. Significant relationships (*P* < 0.05) are marked with an asterisk

Dendroclimatic analyses were performed to detect which climate variables influence the growth trends and wood-anatomical traits. The mean raw and detrended wood-anatomical data are shown in Fig. 1 and Table S2 for the analyses. Growth–climate correlations (i.e., CS traits) showed a positive and significant correlation between BAI and SPEI during the previous growing season (1990–2011) in June for most of the families (Fig. 2). There was also a positive correlation between BAI and SPEI in June of the current growing season, but it was less significant compared to the previous growing season. Radial growth was negatively correlated with higher temperatures in the previous year and positively correlated with higher precipitation during the previous growing season (Fig. S3). Furthermore, LDR and SPEI correlation showed a positive and significant response in July and August of the current growing season (Fig. S4) and WD, CWT, and CWRr revealed negative and significant correlations in July and August of the current growing season (Fig. S4, S5).

### Correlations between drought response, wood-anatomical and growth traits

The genetic and phenotypic correlations between DR, wood-anatomical, and growth traits were examined using GBLUP and ABLUP (Table 2; Table S3). Genetic correlations were generally higher than phenotypic correlations; however, the number of significant correlations was larger for phenotypic correlations in both the GBLUP and ABLUP models (Table 2; Table S3). Using GBLUP, BAI showed significant positive genetic and phenotypic correlations with LDR ($\:\widehat{r}_{a}$_=_ 0.54, *p* < 0.01; $\:\widehat{r}_{p}$_=_ 0.44, *p* < 0.001), HGT ($\:\widehat{r}_{a}$_=_ 0.87, *p* < 0.001; $\:\widehat{r}_{p}$_=_ 0.39, *p* < 0.001), and DBH ($\:\widehat{r}_{a}$_=_ 0.96, *p* < 0.001; $\:\widehat{r}_{p}$_=_ 0.76, *p* < 0.001); and negative correlations with WD ($\:\widehat{r}_{a}$_=_ -0.60, *p* < 0.01; $\:\widehat{r}_{p}$_=_ -0.30, *p* < 0.001) and CWRr ($\:\widehat{r}_{a}$_=_ -0.61, *p* < 0.01; $\:\widehat{r}_{p}$_=_ -0.32, *p* < 0.001) (Table 2). RC had significant genetic and phenotypic correlations with BAI ($\:\widehat{r}_{a}$_=_ 0.91, *p* < 0.01; $\:\widehat{r}_{p}$_=_ 0.32, *p* < 0.001), LDR ($\:\widehat{r}_{a}$_=_ 0.47, *p* < 0.05; $\:\widehat{r}_{p}$_=_ 0.23, *p* < 0.01), HGT ($\:\widehat{r}_{a}$_=_ 0.99, *p* < 0.001; $\:\widehat{r}_{p}$_=_ 0.19, *p* < 0.001), and DBH ($\:\widehat{r}_{a}$_=_ 0.81, *p* < 0.01; $\:\widehat{r}_{p}$_=_ 0.25, *p* < 0.001), whereas RS was correlated only with BAI ($\:\widehat{r}_{a}$_=_ 0.55, *p* < 0.05; $\:\widehat{r}_{p}$_=_ 0.25, *p* < 0.001), and HGT ($\:\widehat{r}_{a}$_=_ 0.68, *p* < 0.01; $\:\widehat{r}_{p}$_=_ 0.07, *p* < 0.05) (Table 2). RL was only phenotypically correlated with LDR $\:\widehat{r}_{p}$_=_ 0.12, *p* < 0.01), HGT ($\:\widehat{r}_{p}$_=_ 0.15, *p* < 0.001), and DBH ($\:\widehat{r}_{p}$_=_ 0.16, *p* < 0.001). HGT and DBH showed negative genetic correlation with WD ($\:\widehat{r}_{a}$_=_ -0.37, *p* < 0.05) and ($\:\widehat{r}_{a}$_=_ -0.61, *p* < 0.001) respectively, whereas the corresponding phenotypic correlations were weak but significant $\:\widehat{r}_{p}$_=_ 0.06, *p* < 0.05) and ($\:\widehat{r}_{p}$_=_ -0.24, *p* < 0.001) (Table 2). Results from GBLUP were comparable to those from ABLUP (Table S3).

**Table 2:** Genetic (above diagonal) and phenotypic (below diagonal) correlations between traits using GBLUP. Narrow-sense heritability estimates are reported on the diagonal (in bold). Standard errors are shown in parentheses along with levels of statistical significance indicated by asterisks: * *p* < 0.05, ** *p* < 0.01, *** *p* < 0.001. Wood-anatomical traits (BAI, LDR, LDT, LD, WD, CWT, CWRr and CWR); growth traits (HGT and DBH); drought response traits (RS, RC and RL)

	BAI	LDR	LDT	LD	WD	CWT	CWRr	CWR	HGT	DBH	RS	RC	RL
BAI	0.27 (0.10)***	0.54 (0.14)**	0.51 (0.19)*	0.59 (0.14)**	-0.60 (0.15)**	-0.35 (0.19)	-0.61 (0.16)**	-0.55 (0.16)**	0.87 (0.04)***	0.96 (0.11)***	0.55 (0.21)*	0.91 (0.19)**	0.51 (0.35)
LDR	0.44 (0.11)***	**0.74 (0.10**)***	0.49 (0.13)**	0.92 (0.03)***	-0.49 (0.11)***	-0.14 (0.14)	-0.65 (0.10)***	-0.52 (0.11)***	0.42 (0.14)**	0.47 (0.13)**	0.20 (0.19)	0.47 (0.22)*	0.45 (0.31)
LDT	0.11 (0.19)**	0.25 (0.17)***	0.48 (0.11)***	0.78 (0.07)***	-0.37 (0.16)*	-0.05 (0.17)	-0.37 (0.17)*	-0.40 (0.16)*	0.63 (0.17)**	0.40 (0.17)*	0.23 (0.23)	0.23 (0.28)	0.20 (0.34)
LD	0.37 (0.12)***	0.84 (0.10)***	0.73 (0.07)***	0.73 (0.10)***	-0.50 (0.12)***	-0.11 (0.15)	-0.60 (0.11)***	-0.53 (0.11)***	0.52 (0.14)**	0.50 (0.13)**	0.25 (0.20)	0.41 (0.24)	0.42 (0.31)
WD	-0.30 (0.11)***	-0.40 (0.17)***	-0.12 (0.14)**	-0.34 (0.18)***	0.57 (0.11)***	0.91 (0.02)***	0.97 (0.01)***	0.99 (0.03)***	-0.37 (0.18)*	-0.61 (0.13)***	0.06 (0.21)	-0.27 (0.24)	-0.42 (0.30)
CWT	-0.17 (0.11)***	-0.11 (0.10)*	0.16 (0.13)***	0.01 (0.18)	0.93 (0.02)***	0.53 (0.11)***	0.81 (0.06)***	0.88 (0.04)***	-0.11 (0.18)	-0.42 (0.17)*	0.17 (0.21)	-0.07 (0.25)	-0.28 (0.31)
CWRr	-0.32 (0.10)***	-0.55 (0.12)***	-0.04 (0.13)*	-0.36 (0.17)***	0.92 (0.02)***	0.85 (0.03)***	0.48 (0.11)***	0.97 (0.09)***	-0.39 (0.18)*	-0.56 (0.1)**	-0.08 (0.23)	-0.36 (0.24)	-0.56 (0.31)
CWR	-0.29 (0.10)***	-0.44 (0.15)***	-0.11 (0.10)*	-0.36 (0.17)***	0.97 (0.01)***	0.89 (0.03)***	0.96 (0.09)***	0.52 (0.10)***	-0.39 (0.18)*	-0.55 (0.15)**	-0.01 (0.22)	-0.25 (0.24)	-0.01 (0.23)
HGT	0.39 (0.10)***	0.23 (0.12)***	0.05 (0.11)*	0.19 (0.14)***	0.06 (0.03)*	0.12 (0.12)**	-0.01 (0.12)	0.04 (0.12)*	0.43 (0.10)***	0.67 (0.13)***	0.68 (0.20)**	0.99 (0.00)***	0.85 (0.43)
DBH	0.76 (0.04)***	0.43 (0.12)***	0.13 (0.12)**	0.37 (0.14)***	-0.24 (0.14)***	-0.11 (0.10)**	-0.28 (0.11)***	-0.25 (0.12)***	0.51 (0.08)***	0.37 (0.10)***	0.38 (0.22)	0.81 (0.19)**	0.42 (0.32)
RS	0.25 (0.08)***	0.18 (0.13)***	0.08 (0.10)	0.17 (0.13)***	-0.06 (0.11)	0.02 (0.10)	-0.09 (0.09)	-0.08 (0.10)	0.07 (0.09)*	0.15 (0.09)***	0.22 (0.09)***	0.71 (0.24)*	0.91 (0.23)*
RC	0.32 (0.07)***	0.23 (0.12)**	0.01 (0.09)	0.17 (0.12)***	-0.11 (0.10)*	-0.05 (0.09)	-0.16 (0.08)***	-0.11 (0.09)*	0.19 (0.08)***	0.25 (0.07)***	0.28 (0.07)***	0.15 (0.08)**	0.90 (0.25)*
RL	0.22 (0.10)	0.12 (0.11)**	0.08 (0.08)	0.13 (0.11)**	-0.01 (0.10)	0.04 (0.09)	-0.03 (0.09)	-0.01 (0.09)	0.15 (0.07)***	0.16 (0.08)***	0.49 (0.05)***	0.54 (0.05)***	**0.07 (0.06)**

### Heritability estimates

Narrow-sense heritability estimates from GBLUP for all the traits were higher than those based on ABLUP (Table 2; Table S3). Heritability for RL was not significant in either GBLUP or ABLUP, as shown by the overlapping standard errors (Table 2; Table S3). Heritability estimates indicated that the traits were under varying genetic control, from high to low, with LDR showing the highest values. Heritability ranged from 0.07 (RL) to 0.74 (LDR) using GBLUP and from 0.05 (RL) to 0.59 (LDR) using ABLUP (Table 2; Table S3). BAI had the lowest heritability (0.14–0.27) compared to other wood-anatomical traits in both ABLUP and GBLUP. All DR traits exhibited lower heritability estimates compared to growth and wood-anatomical traits. Heritability estimates for wood-anatomical traits were substantially higher than for growth traits in both GBLUP and ABLUP. Wood characteristics related to cell anatomy such as LDR (0.74–0.59), CWT (0.53–0.37), CWRr (0.48–0.32), and LDT (0.48–0.26) were under strong to moderate genetic control, whereas growth traits like HGT (0.43–0.22) and DBH (0.37–0.23) exhibited only moderate heritability (Table 2; Table S3).

### Prediction efficiency methods for the ABLUP and GBLUP

**Fig. 3:**
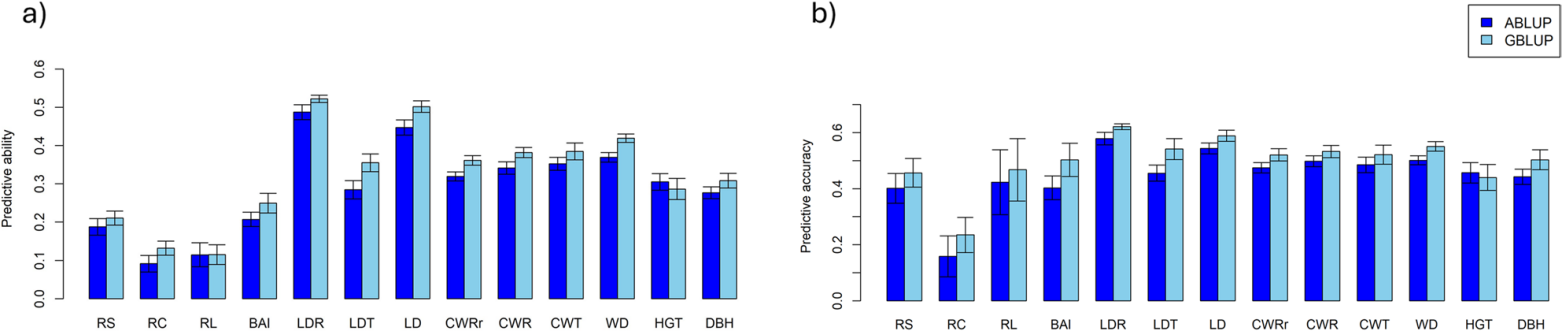
Prediction efficiencies of GBLUP and ABLUP for phenotypic traits: (**a**) predictive ability (PA), and (**b**) predictive accuracy (PAC). Error bars on the histograms represent standard errors. Wood-anatomical traits (BAI, LDR, LDT, LD, WD, CWT, CWRr and CWR); growth traits (HGT and DBH); drought response traits (RS, RC and RL)

Prediction efficiency for both ABLUP and GBLUP used to estimate PA and PAC was assessed using a ten‑fold CV scheme (Fig. 3). GBLUP showed better estimation of prediction efficiencies of PA and PAC for almost all the traits. The PA and PAC values in either GBLUP or ABLUP models were larger for the traits with higher heritability, e.g., wood-anatomical and growth traits, compared to DR traits with low heritability (Fig. 3; Table 2). In terms of predictive estimation, the GBLUP model showed that the PA ranged from 0.12 for RL to 0.52 for LDR. The ABLUP model showed a comparable pattern, with PA values ranging from 0.10 for RC to 0.49 for LDR. The PAC was moderate for the DR traits in both models 0.23–0.47 in GBLUP, and 0.16–0.42 in ABLUP, while wood anatomical traits exhibited higher PAC values, reaching 0.50–0.61 in GBLUP and 0.41–0.57 in ABLUP (Fig. 3).

### ABLUP and GBLUP accuracy

To ensure comparability of GBLUP validation results, ABLUP accuracy (TAC) were estimated without applying any CV procedure using all the phenotypic data. Such an approach enables a meaningful comparison of whether genomic prediction without phenotypic information can achieve accuracy comparable to predictions based solely on phenotypes. Across traits, GBLUP consistently had comparable theoretical accuracies with ABLUP (Table 3). For TAC, GBLUP showed better accuracy for DR (0.54–0.62) and growth traits (0.66–0.67), indicating an increased expected reliability due to the incorporation of genomic relationships, whereas ABLUP showed better accuracy for wood‑anatomical traits (0.58–0.82) (Table 3).

**Table 3:** Theoretical accuracy of GBLUP and ABLUP for phenotypic traits. GBLUP_CV: accuracy estimated applying cross‑validation procedure. ABLUP_without_CV: accuracy estimated without applying cross‑validation. Error bars are shown in parentheses

Traits	GBLUP_CV	ABLUP_without_CV
RS	0.62 (0.003)	0.54 (0.002)
RC	0.59 (0.008)	0.38 (0.001)
RL	0.54 (0.009)	0.39 (0.001)
BAI	0.64 (0.004)	0.58 (0.002)
LDR	0.71 (0.003)	0.82 (0.001)
LDT	0.68 (0.004)	0.67 (0.002)
LD	0.70 (0.003)	0.79 (0.001)
CWRr	0.68 (0.003)	0.70 (0.002)
CWR	0.68 (0.003)	0.72 (0.001)
CWT	0.68 (0.003)	0.73 (0.002)
WD	0.69 (0.003)	0.73 (0.001)
HGT	0.67 (0.005)	0.64 (0.002)
DBH	0.66 (0.003)	0.64 (0.002)

### Relative response to GS

The estimated relative response to genomic selection (*RSR*_*GS*:*PS*_) for TAC showed an increase in genetic gain for all traits. For backward selection, a 50% reduction in the breeding cycle (from 36 to 18 years) resulted in 74.2%–211.6% increase in genetic gain (Table 4). A further reduction in the breeding cycle (from 36 to 11 years) increased the gain to 185%–410%. For forward selection, a three-year reduction (21 to 18 years) in the breeding cycle resulted in only a small genetic gain (1.6% to 81.8%), indicating that the traditional phenotypic selection would be more efficient for some traits where low gains were obtained (Table 4). A ~ 50% reduction in the breeding cycle (from 21 to 11 years) increased the gain between 66.3% and 197.5% for TAC. For all traits, the increase in genetic gain exceeded 50% when the breeding cycle was reduced by 50% (Table 4).

**Table 4:** Percentage increase in genomic selection efficiency for each phenotypic trait, estimated for both backward and forward selection strategies using GBLUP to ABLUP ratio in theoretical accuracy (TAC). Wood-anatomical traits (BAI, LDR, LDT, LD, WD, CWT, CWRr and CWR); growth traits (HGT and DBH); drought response traits (RS, RC and RL)

Strategy	Prediction efficiency	Model ratio	RS	RC	RL	BAI	LDR	LDT	LD	CWRr	CWR	CWT	WD	HGT	DBH
Backward selection reduction of 36 to 18 years	TAC	GBLUP/ABLUP	128.3	211.6	174.4	120.7	74.2	103.6	77.2	94.3	89.4	86.8	87.9	108.1	104.8
Backward selection reduction of 36 to 11 years	TAC	GBLUP/ABLUP	273.5	410.0	349.0	261.1	185.0	233.2	190.0	217.9	209.9	205.7	207.5	240.6	235.1
Forward selection reduction of 21 to 18 years	TAC	GBLUP/ABLUP	33.1	81.8	60.1	28.7	1.6	18.8	3.4	13.4	10.5	9.0	9.6	21.4	19.5
Forward selection reduction of 21 to 11 years	TAC	GBLUP/ABLUP	117.9	197.5	161.9	110.7	66.3	94.4	69.2	85.4	80.8	78.3	79.4	98.7	95.5

## Discussion

### Genetic variation for drought response traits among families

We found substantial genetic variation for the DR traits in response to drought among families (Table 1; Fig. S2). During the drought period, the most resistant families had the highest BAI growth and exhibited better resilience but recovered less in the following year. Most of the families with weaker radial growth during drought episodes showed lower resistance to drought but recovered better afterwards (Fig. S2). This trade-off between resistance and recovery after drought episodes has also been observed in many conifer tree species, where trees with higher resistance revealed a lower recovery rate [1, 18, 34, 92, 93]. Carbon reserves stored in the trees are reported to be a determining factor for recovery [46, 94]. The ability of trees to survive and recover from drought episodes depends on the balance between carbon gain and water loss [95]. Low resilience in most of the families could be due to an effective adaptive strategy that reduces growth under drought conditions but avoids structural damage, thereby supporting long-term productivity [18]. Reduced radial growth under drought may thus represent a universal adaptive strategy that enhances survival across all families, regardless of its genetic background [96, 97].

### Growth–climate correlations across tree lifespan (i.e. climate-sensitivity traits)

The climate–growth relationships showed variation among families, which was also reported in white spruce [22]. We found that SPEI was highly correlated with BAI and explained a significant portion of growth variation in Scots pine (Figs. 1 and 2). Similar results were reported in previous studies on drought stress and growth reduction in white spruce [19, 22, 37]. Temperature showed a negative impact on the radial growth of Scots pine, while precipitation and SPEI in the previous growing season had significant and positive effects on the radial growth (Fig. 2; Fig. S3). However, some families had positive and significant effect in the current growing season for SPEI and precipitation due to the carry-over effects [98–100], a phenomenon in which nutrition storage such as carbohydrates and water reserves from the previous year are used in the following year for growth. This finding is in line with previous studies [37, 101–103]. Furthermore, LDR and SPEI correlation showed positive responses in July and August of the current growing season, whereas correlations of WD, CWT and CWRr with SPEI revealed negative responses in July and August of the current growing season (Fig. S4; Fig. S5). Conifers exposed to prolonged drought have been characterized by the adaptation in xylem structure, such as a large LDR, which can improve total hydraulic conductivity without extra carbon costs [104].

Forest trees use different adaptive strategies to cope with severe droughts. Reduced radial growth in a drought period may result from reduced photosynthetic activity due to partial stomatal closure to maintain xylem water potentials, as well as reduced allocation of carbohydrates to radial growth due to enhanced investment in roots [96, 105]. This process reduces the risk of cavitation but may increase risk of hydraulic failure [106, 107], indicating a trade-off between hydraulic conductivity and cavitation resistance [39]. Reduced radial growth could also reflect an acclimation response, where a tree survives drought by producing more cavitation-resistant xylem with tracheids that have thicker cell walls and smaller lumen diameters. This helps to avoid depletion of carbon reserves by keeping stomata open under prolonged drought [106], reducing hydraulic efficiency but increasing hydraulic safety.

### Genetic and phenotypic correlations between traits

With the rapid climatic change, tree breeders need strategies to incorporate DR traits into breeding programmes while maintaining productivity. Positive genetic and phenotypic correlations were observed between DR, growth, and wood-anatomical traits (BAI and LDR) (Table 2; Table S3) in our study, which is in agreement with earlier studies [22, 108]. This indicates that mature and vigorous trees during drought periods tend to develop adequate root systems and resist stress better by allocating carbon reserves differently to roots [109]. All DR traits were positively correlated with BAI (Table 2; Table S3). There was no trade-off between increased DR traits and BAI, suggesting that breeding for improved DR traits would not hamper growth quality in future wood plantations. Similar results were shown in white spruce [19], where all DR traits except for the resistance were correlated with BAI. Although some fast-growing families showed weaker drought recovery in our dataset, this reflects phenotypic variation rather than a genetic trade-off, and therefore should not constrain long-term selection for increased growth. Drought resistance is often presumed to enhance long‑term survival, yet this link is not always clear in conifers. Trees with comparatively lower drought resistance but higher growth rates may achieve better long-term survival simply because their faster growth enhances competitive success [110, 111]. However, breeding programs often select for genotypes that maintain relatively high growth rates even during drought conditions, which makes them appear drought tolerant. Thus, selection of drought tolerance trees in forestry primarily reflects sustained growth performance during drought periods rather than high physiological drought resistance. Wood density was negatively correlated with DR and growth traits, showing it to be poor predictor of DR and growth performance (Table 2; Table S3). These correlation patterns align with results from other studies in conifers [22, 44, 108]. Furthermore, some studies have revealed a trade-off between growth and resistance to abiotic stress in conifer trees [39, 112]. Practically, breeders need to integrate multiple traits simultaneously for selection like growth and wood quality in addition to pest, pathogen and drought resistant traits to accelerate breeding [23]. A multi-trait model may show improvement in predictive accuracy of a target trait of low heritability and traits that are costly or difficult to evaluate [22, 44, 45]. Several studies have emphasized that multi-trait models can improve the prediction accuracy of a target trait when it is analysed jointly with genetically correlated indicator traits that exhibit high heritability. In such models, each trait contributes information about the genetic values of the others, thereby enhancing overall accuracy [44, 113, 114]. However, the adverse correlation between WD, which is responsible for mechanical strength, and wood-anatomical traits related to cell anatomy as well as growth traits, could suggest a need for careful selection strategies to balance trade-offs between mechanical strength and pulp quality in the forest industry [27, 115, 116].

### Heritability estimates

Our results for Scots pine showed moderate to high heritability estimates for wood-anatomical and growth traits (Table 2; Table S3), similar to other conifer species [19, 26, 53, 57, 115, 116]. In contrast, heritability estimates for DR traits were low to moderate (Table 2; Table S3), and lower than those reported in white spruce [19, 22, 108]. In our study, RS was the most heritable trait, followed by RC among the DR traits during the drought period in mature progeny trial, which contrasted with observations in white spruce [19]. However, higher heritability for RS was found by Laverdière et al. [22] at one site where more severe drought affected current growth, indicating that greater genetic gains could be obtained under severe drought events. We expect that these traits have the potential to improve the resilience of trees under drought conditions. The reported narrow-sense heritability estimates in Scots pine for DBH, HGT and WD using GBLUP were higher than those previously reported by Calleja-Rodriguez et al. [26] in a Scots pine progeny trial. This might be expected, as GBS reads in our study were mapped to the Scots pine reference genome (under review) instead of loblolly pine (*P. taeda* L.) v1.0 reference genome to detect SNPs [62], with 31,101 SNPs used, compared to 8,719 SNPs used in the previous study [26]. Heritability estimates for growth traits in our study were also higher than those reported for other conifer species [27, 117], but lower than those reported by Lenz et al. [59]. The heritability of wood-anatomical traits was larger in our study compared to other studies in conifers [19, 108, 116]. Given the positive genetic correlation and moderate to high heritability, LDR and BAI, apart from HGT and DBH, appear to be the most suitable candidate traits for genetic improvement.

### Predictive efficiency of GS models

In previous studies, different genomic statistical methods were used to estimate prediction efficiencies. However, these methods did not clearly outperform each other and produced similar results for growth and wood quality traits in other forest tree species [26, 27, 58]. GBLUP has been found to be an effective method for estimating prediction efficiencies [27]. In our study, GBLUP performed slightly better than ABLUP in terms of PA and PAC (Fig. 3a, b). As GBLUP accounts for Mendelian segregation within families, in contrast to ABLUP, we expect more accurate estimates of relatedness between individuals by relying on their genomic profiles [118]. Our results are in alignment with previous findings for forest trees, where GBLUP prediction accuracy was slightly higher than ABLUP [43, 85]. On the contrary ABLUP having prediction accuracy higher than GBLUP model have been reported in other studies [26, 57]. We found that the PA and PAC for DR traits were lower than those for wood-anatomical and growth traits and were comparable between GBLUP and ABLUP (Fig. 3a, b). We obtained low to medium PAC for all DR traits studied (Fig. 3b), consistent with results reported in white spruce [22]. PA and PAC of wood-anatomical traits were higher compared to the observations in white spruce [57]. The precision of the PAC may be affected by the low precision of heritability estimates and other factors [44, 119]. In a CV step, ABLUP models lack an ability to dissect the Mendelian-segregation effects within family, thus, theoretically its prediction efficiency should be lower than GBLUP without using within-family phenotypes. Since we have phenotypic data for all trees including validation population, the breeding value accuracy for validation population in the ABLUP models should use estimated or theoretical accuracy from the ABLUP models, not the breeding value accuracy calculated using pedigree relationship only without the phenotype for the validation population. Therefore, we suggest that CV using pedigree relationship only without the phenotype to estimate ABLUP accuracy for the validation population is invalid to compare (or evaluate) the efficiency of GBLUP in GS relative to traditional phenotypic selection.

### Accurate evaluation of GBLUP efficiency compared to traditional ABLUP selection

To compare the efficiency of GBLUP with traditional ABLUP selection, the TAC was calculated differently for each method. GBLUP prediction accuracy was evaluated through CV, whereas ABLUP accuracy was determined analytically using pedigree information. For TAC, GBLUP showed better accuracy for DR and growth traits highlighting the advantage of genomic selection in capturing within-family Mendelian sampling, whereas ABLUP showed better accuracy for most of the wood‑anatomical traits showing ABLUP without CV uses all the phenotypes to estimate EBVs thereby reducing prediction error variance. This pattern is consistent with previous tree breeding studies showing that genomic models provide greater benefits for low‑heritability, polygenic traits such as growth, while pedigree‑based models often perform comparably or better for moderately to highly heritable wood and wood‑anatomical traits due to strong family structure [22, 27, 57]. In tree breeding, avoiding CV in pedigree-based ABLUP prevents biases caused by strong family structure and relatedness across folds, even though it yields optimistic accuracy estimates and limits direct comparison with cross-validated genomic models [60, 119]. This method cannot replace phenotypic ABLUP prediction nor serve as a direct comparator to cross-validated genomic selection accuracy.

### Efficiency of genomic selection

The efficiency of genomic selection relative to phenotypic selection was evaluated using theoretical accuracies of breeding values. Phenotypic selection accuracy was represented by expected EBV accuracies from an ABLUP model fitted using full pedigree and phenotypic information, while genomic selection accuracy was assessed using theoretical GEBV accuracies from GBLUP. In the current study, we found that across traits the efficiency of GS was higher than PS (pedigree-based selection) when generation time was reduced to half (Table 4). The results showed that a 50% reduction in the breeding cycle length doubled the efficiency of GS over PS for DR and wood-anatomical traits, and a 75% reduction tripled the efficiency indicating a higher expected response to selection when genomic information is used. These findings are consistent with previous studies in conifers for growth traits [26, 27, 89, 119]. With the rapid decrease in genotyping costs, genotyping is becoming cheaper than establishing, maintaining, and testing progenies in field trials. GS would be highly beneficial, as selection can be performed at the seedling stage, thereby shortening the generation intervals and reducing the cost and time required for progeny testing in the field. Individuals identified at the early seedling stage through GS could also be propagated by somatic embryogenesis or rooted cuttings and mass-produced for large-scale reforestation programmes within only a few years [54]. With a forward selection scheme, the breeding cycle could be reduced substantially while increasing genetic gain per unit time [59]. In the face of drought-prone environments, DR traits may be incorporated into breeding strategies targeted for improved growth performance. GS has the potential to reduce the costs associated with maintaining long-term progeny trials and extensive phenotyping, while also enabling higher selection intensity. However, GS alone does not shorten the generation interval. Therefore, it is important to develop robust strategies to evaluate the economic efficiency of genomic enabled breeding relative to traditional phenotypic selection.

## Conclusion

As temperatures rise and precipitation patterns shift due to climate change, identifying tree genotypes (resilient provenances and families) that can survive under increasing drought conditions across different environments is essential for the future. Long-term progeny trials provide the opportunity to assess drought resistance using annual ring patterns formed during past drought episodes. This study demonstrates that combining dendroecology and quantitative genetics approaches helps us understand genetic variation in drought resistance-related traits. We found that:

1. Precipitation and SPEI in the previous growing season had positive effects on the radial growth.
2. There is significant genetic variation in drought resistance traits - resistance, recovery, and resilience.
3. Heritability for drought resistance traits was lower than for growth traits, whereas wood-anatomical traits and wood density had the highest heritabilities.
4. Genomic selection had similar estimation as pedigree-based selection for drought resistance traits.
5. Positive genetic correlations between drought resistance traits and growth traits enable simultaneous selection for higher growth and improved drought resistance in Scots pine breeding.

## Supplementary Information

Supplementary Material 1

## Data Availability

The data-sets used and/or analyzed during the current study are available from Skogforsk and the corresponding author on reasonable request for research purposes.

## Abbreviations

ABLUP: Pedigree-based best linear unbiased predictions
A matrix: Pedigree-based relationship matrix
AR: Autoregressive
BAI: Basal area increment
CL_GS_: Breeding cycle lengths of Genomic selection
CL_PS_: Breeding cycle lengths of Pedigree-based selection
CS traits: Climate-sensitivity traits
CV: Cross-validation
CWRr: Radial conduit wall reinforcement
CWR: Conduit-wall reinforcement
CWT: Cell wall thickness
DBH: Diameter at breast height
DR traits: Drought-response traits
EBVs: Pedigree-based breeding values
GBS: Genotyping-by-sequencing
GBLUP: Genomic best linear unbiased predictions
GEBVs: Genomic estimated breeding values
G matrix: Genomic relationship matrix
GS: Genomic selection
HGT: Tree height
LD: Mean lumen diameter
LDR: Radial lumen diameter
LDT: Tangential lumen diameter
MAF: Minor allele frequency
MAP: Mean annual precipitation
MAT: Mean annual temperature
MFA: Microfibril angle
MOE: Modulus of elasticityPA: Predictive ability
PAC: Predictive accuracy
PRA: Prediction accuracy
PS: Pedigree-based selection
$$\:\widehat{r}_{a}$$: Genetic correlations
RC: Recovery
r_GS_: Efficiency of Genomic selection
RL: Resilience
$$\:\widehat{r}_{p}$$: Phenotypic correlations
r_PS_: Efficiency of Pedigree-based selection
RS: Resistance
RSR: Relative selection response
RW: Annual ring-widths
SNPs: Single nucleotide polymorphisms
SPEI: Standardised potential evapotranspiration index
SPI: Standardized precipitation index
TAC: Theoretical accuracy
TP: Training population
VP: Validation population
WD: Wood density
