# Supplementary material for: Breeding for climate adaptation: genetic variation and genomic selection for drought response in Scots pine
Source: BMC Genomics. 2026 Apr 27;27:416. doi: 10.1186/s12864-026-12849-x (PMC13122877; doi:10.1186/s12864-026-12849-x)
Supplement: Supplementary file 1 — Supplementary Material 1 [file 12864_2026_12849_MOESM1_ESM.zip › additional_files/supplementary_figures.pdf]

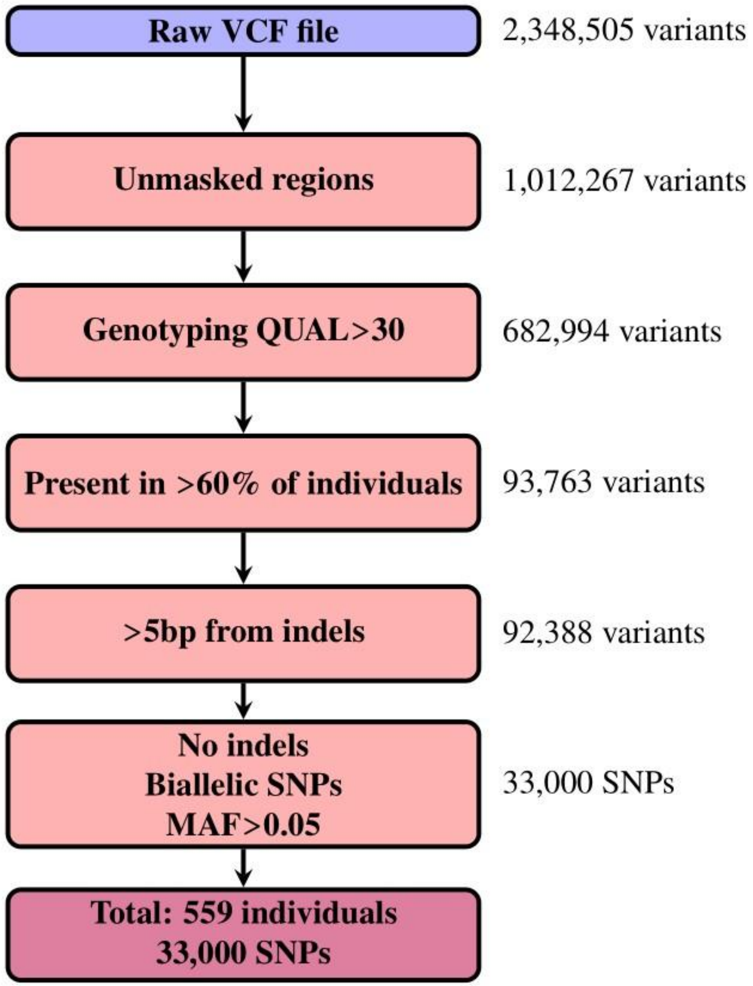

Fig. S1: Flow chart of the variant filtering process.

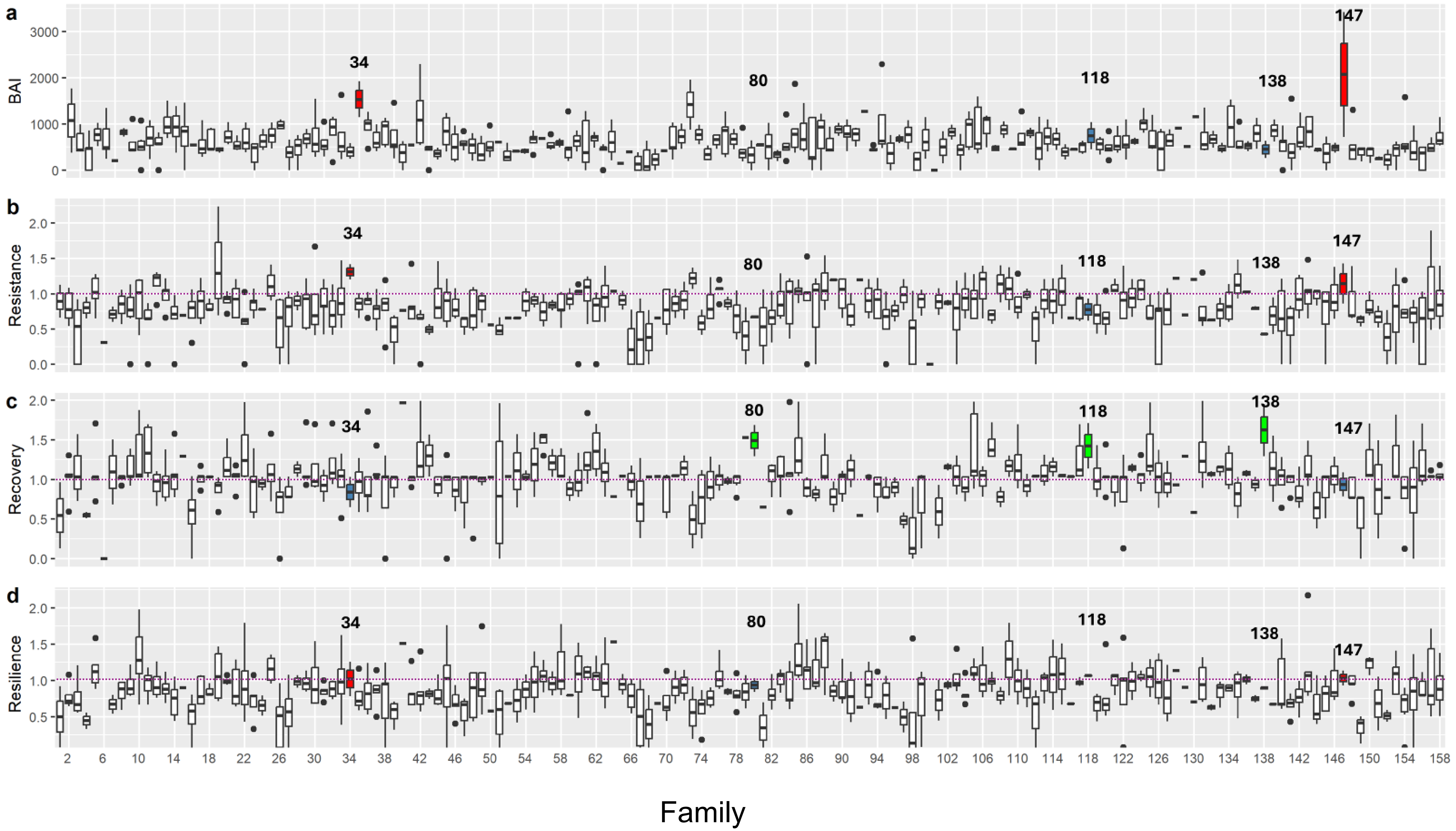

a
BAI
b
Resistance
c
Recovery
d
Resilience
34
80
118
138
147
Family

Fig. S2: The performance of Scots pine families at the Grundtjärn site in drought period (2002-2003). The box plot shows two lines at the 25th percentile and 75th percentile. The intermediate line is the median within the box. The whiskers represent the variability outside the upper and lower quartiles. Dots shows outliers. The best-performing families (family 34 and 147) are shown in red colour, i.e., high resistance and BAI growth with better resilience. The families with higher recovery rate (family 80, 118 and 138) are indicated in green colour, which shows weaker BAI growth, as well as lower resistance and resilience indicated in blue colour. The dash purple line shows average threshold for resistance, recovery, and resilience. For resistance and resilience, values of 1 indicate no decline in increment or an immediate return to pre-drought level, respectively, and for recovery values >1 suggest an immediate regeneration from a drought event.

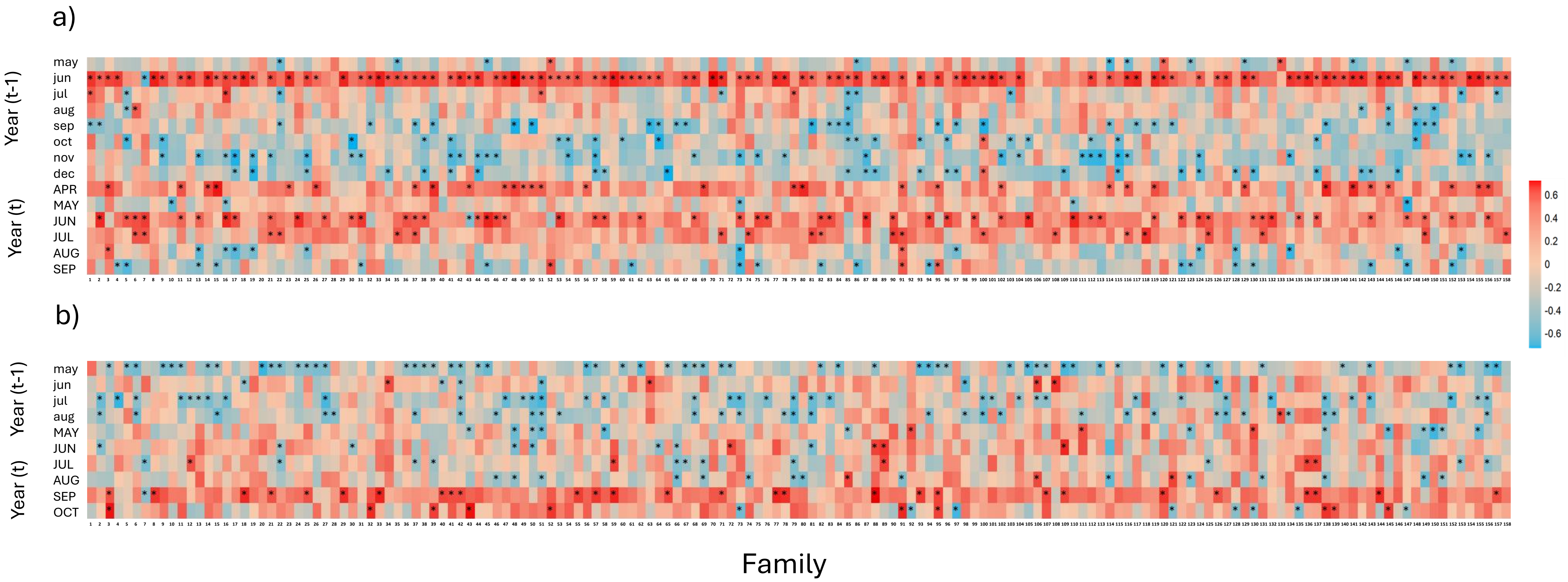

Fig. S3: Climate–growth associations of Scots pine families. (a) Correlation analyses of BAI residual chronologies and monthly total precipitation at the Grundtjärn site from May to December of the previous growing season (t − 1) and April to September of the current growing season (t) over the 1990 –2011 period. (b) Correlation analyses of BAI residual chronologies and monthly mean temperature from May to August of the previous growing season (t − 1) and May to October of the current growing season (t). Months in capital letters represent the current year of ring formation, and months in lower case represent the preceding year ring formation. The scale bar indicates positive (red) and negative (blue) correlation coefficients. Significant relationships (*P* < *0.05*) are marked with an asterisk.

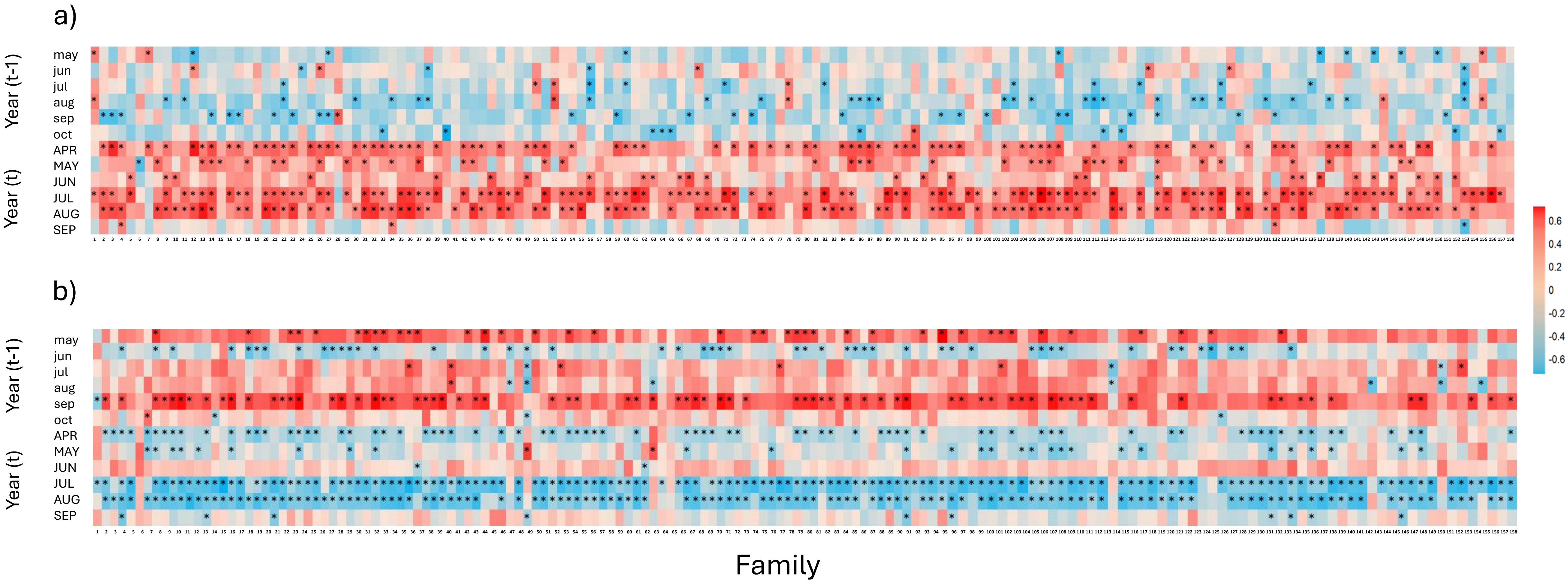

Fig. S4: Climate–growth associations of Scots pine families. (a) Correlation analyses of LDR residual chronologies and monthly SPEI from May to October of the previous growing season (t − 1) and April to September of the current growing season (t). (b) Correlation analyses of WD residual chronologies and monthly SPEI from May to October of the previous growing season (t − 1) and April to September of the current growing season (t). Months in capital letters represent the current year of ring formation, and months in lower case represent the preceding year ring formation. The scale bar indicates positive (red) and negative (blue) correlation coefficients. Significant relationships (*P* < *0.05*) are marked with an asterisk.

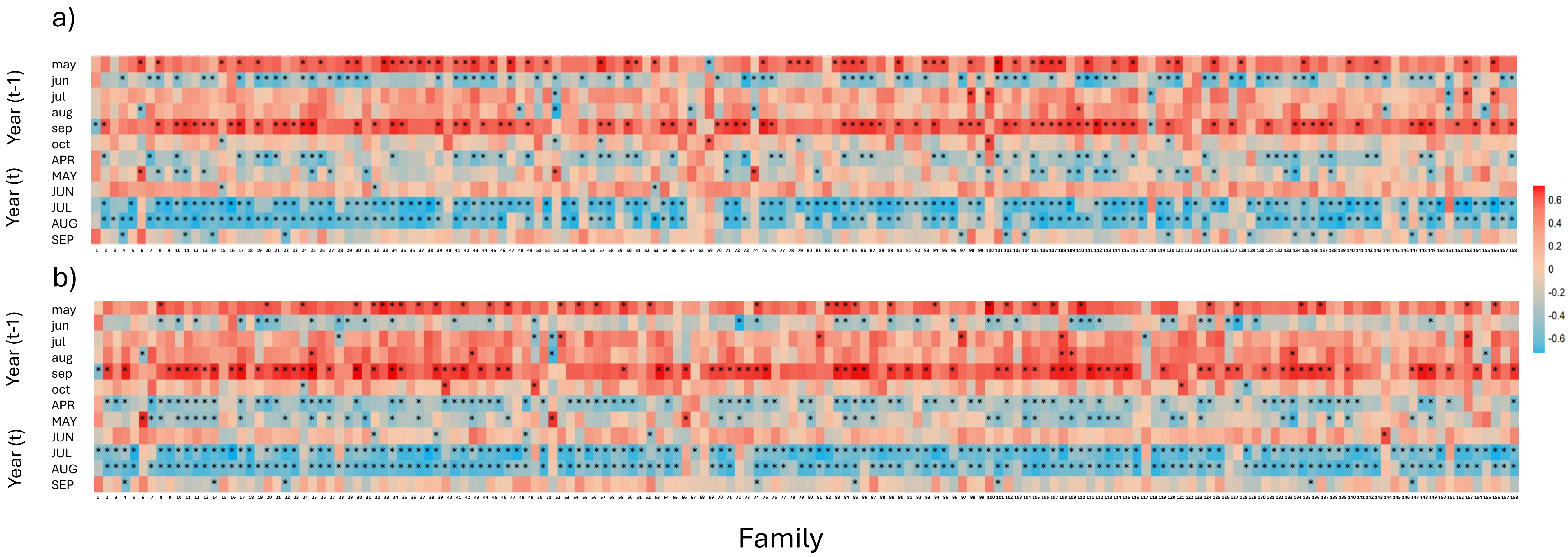

Fig. S5: Climate–growth associations of Scots pine families. (a) Correlation analyses of CWT residual chronologies and monthly SPEI from May to October of the previous growing season (t − 1) and April to September of the current growing season (t). (b) Correlation analyses of CWRr residual chronologies and monthly SPEI from May to October of the previous growing season (t − 1) and April to September of the current growing season (t). Months in capital letters represent the current year of ring formation and months in lower case represent the preceding year ring formation. The scale bar indicates positive (red) and negative (blue) correlation coefficients. Significant relationships ($P < 0.05$) are marked with an asterisk.
