# Supplementary material for: Breeding for climate adaptation: genetic variation and genomic selection for drought response in Scots pine
Source: BMC Genomics. 2026 Apr 27;27:416. doi: 10.1186/s12864-026-12849-x (PMC13122877; doi:10.1186/s12864-026-12849-x)
Supplement: Supplementary file 1 — Supplementary Material 1 [file 12864_2026_12849_MOESM1_ESM.zip › additional_files/supplementary_mehodS1.pdf]

Method S1: Calculation details of drought and drought response index.

Calculation of drought index: SPEI (standardised potential evapotranspiration index) and SPI (standardized precipitation index) are calculated using the "SPEI" package (Beguería & Vicente-Serrano, 2017).

The SPI calculates the precipitation data linked to the selected probability distribution and is further standardized using the normal distribution with zero mean and standard deviation of one. The SPI probability distribution was calculated using the Gamma distribution.

The SPEI integrates both the precipitation and potential evapotranspiration data. SPEI is calculated using the differences between precipitation and potential evapotranspiration. This represents a simple climatic water balance, which is calculated at different time scales to obtain the SPEI. The probability distribution of SPEI was expressed using the three-parameter (α, β and γ) log-logistic probability distribution with parameters fitted using unbiased probability weighed moments (Vicente-Serrano et al., 2010).

Calculation of drought response index: Drought response indexes of the tree were developed by Lloret et al. (2011)

Resistance (RS): the capacity of trees to withstand drought and resist growth reduction. RS was estimated as DR/preDR, where DR and preDR are the ratio between BAI during and before the drought period, respectively.

Recovery (RC): the ability of a tree to recover its growth after drought period. RC was estimated as postDR/DR, where postDR and DR are the ratio between BAI after and during the drought period, respectively.

Resilience (RL): the ability of tree to reach pre-drought growth levels after drought. RS was calculated as postDR/preDR.
